# Pristimerin, a Triterpenoid, Inhibits Tumor Angiogenesis by Targeting VEGFR2 Activation

**DOI:** 10.3390/molecules17066854

**Published:** 2012-06-05

**Authors:** Xianmin Mu, Wei Shi, Lixin Sun, Han Li, Zhenzhou Jiang, Luyong Zhang

**Affiliations:** 1 Jiangsu Center for Drug Screening, China Pharmaceutical University, Nanjing 210009, Jiangsu, China; Email: cpumxm@hotmail.com (X.M.); slxcpu@126.com (L.S.); lihan@hotmail.com (H.L.); 2 The Secondary Affiliated Hospital of Nanjing Medical University, Nanjing 210011, Jiangsu, China; 3 Department of New Drug Screening, Jiangsu Chia-tai Tianqing Pharmaceutical Co., Ltd, Nanjing 210042, Jiangsu, China; Email: shiwei411@hotmail.com; 4 Key Laboratory of Drug Quality Control and Pharmacovigilance, Ministry of Education, Nanjing 210009, Jiangsu, China

**Keywords:** pristimerin, angiogenesis, cancer, KDR/Flk-1, AKT/mTOR

## Abstract

Pristimerin is a triterpenoid isolated from *Celastrus* and *Maytenus spp.* thathas been shown to possess a variety of biological activities, including anti-cancer activity. However, little is known about pristimerin’s effects on tumor angiogenesis. In this study, we examined the function and the mechanism of this compound in tumor angiogenesisusing multiple angiogenesis assays. We found that pristimerin significantly reduced both the volume and weight of solid tumors and decreased angiogenesis in a xenograft mouse tumor model *in vivo*. Pristimerin significantly inhibited the neovascularization of chicken chorioallantoic membrane (CAM) *in vivo* and abrogated vascular endothelial growth factor (VEGF)-induced microvessel sprouting in an *ex vivo* rat aortic ring assay. Furthermore, pristimerin inhibited the VEGF-induced proliferation, migration and capillary-like structure formation of human umbilical vascular endothelial cells (HUVECs) in a concentration-dependent manner. Mechanistic studies revealed that pristimerin suppressed the VEGF-induced phosphorylation of VEGF receptor 2 kinase (KDR/Flk-1) and the activity of AKT, ERK1/2, mTOR, and ribosomal protein S6 kinase. Taken together, our results provide evidence for the first time that pristimerin potently suppresses angiogenesis by targeting VEGFR2 activation. These results provide a novel mechanism of action for pristimerin which may be important in the treatment of cancer.

## 1. Introduction

Tumor angiogenesis, that is, the development of new blood vessels from pre-existing ones, plays an essential role in tumor growth, invasiveness, and metastasis [1,2]. It is estimated that angiogenesis in tumors contributes to more than 90% of all cancer deaths. Tumor angiogenesis is a promising therapeutic target for treatment of cancer. Angiogenesis is initiated by cell proliferation and migration in response to chemotactic agents, such as VEGF, which is widely expressed in the majority of cancers and is a critical component of tumor angiogenesis [3]. VEGF mediates its signals through interactions with the receptor tyrosine kinases expressed on endothelial cells [4]. VEGFRs activation leads to the activation of diverse intracellular signaling molecules, including extracellular signal-regulated kinases (ERKs), phosphoinositide 3-kinase/AKT kinase, and mammalian target of rapamycin (mTOR)/ribosomal protein S6 kinase (p70S6K) [5,6] that promote the proliferation, migration, differentiation, and survival of endothelial cells in the pre-existing vasculature. Thus, VEGFR2 and those intracellular signaling molecules appear to be critical targets for the suppression of tumor angiogenesis. Multiple angiogenesis inhibitors have been therapeutically validated in treatments for many cancers [7].

**Figure 1:**
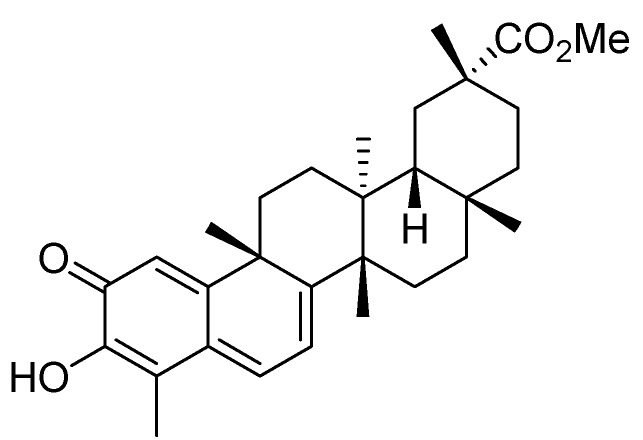
Chemical structure of pristimerin.

Pristimerin (Figure 1) is a natural triterpenoid isolated from Celastrus and Maytenus spp. [8] that has been shown to possess a variety of biological activities. Antitumor properties have also been reported for pristimerin. Previous studies have shown that pristimerin can inhibit tumor cell proliferation by inhibiting the NF-κB pathway and cell cycling [8,9,10,11,12]. In addition, pristimerin has been reported to induce caspase-dependent apoptosis of breast cancer cells [13] and prostate cancer cells *in vitro* [11]. Recently, pristimerin is reported to inhibit xenografted plasmacytoma tumors in mice through the suppression of 20s proteasome chymotrypsin-like activity [9]. Despite reports of the antitumor properties of pristimerin, whether this compound directly affects tumor angiogenesis or has potential value for breast cancer prevention *in vivo* remains unknown.

In this study, we investigated how pristimerin inhibits tumor angiogenesis by targeting key signaling pathways. Our results provide the first evidence that pristimerin significantly inhibits VEGF-stimulated endothelial cell proliferation, migration, tube formation, and tumor angiogenesis by targeting VEGFR2 activation, leading to the suppression of tumor growth.

## 2. Results and Discussion

### 2.1. Pristimerin Inhibits Tumor Growth and Tumor Angiogenesis in a Xenograft Mouse Model

**Figure 2:**
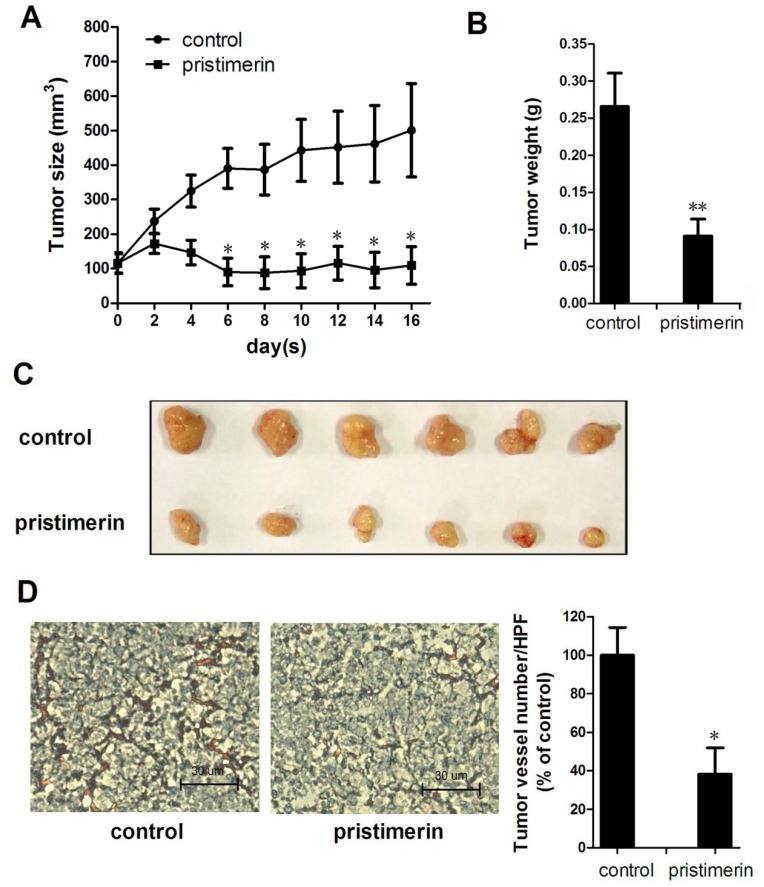
pristimerin inhibits tumor growth and tumor angiogenesis in xenograft mice. MDA-MB-231 cells were injected into Six week-old female BALB/c nude mice (8 × 10^6^ per mouse). After solid tumors grew to about 100 mm^3^, mice were s.c. given with or without pristimerin (3 mg/kg every other day). (**A**) pristimerin inhibited tumor growth as measured by tumor volume; (**B/C**) solid tumors in the pristimerin-treated mice were significantly smaller than in the untreated mice; (**D**) blood vessel staining revealed that pristimerin inhibited tumor angiogenesis (magnification, ×200). Arrows, blood vessels. Columns, mean; bars, SD. * *p* < 0.05 or ** *p* < 0.01 *versus* control.

To investigate the effects of pristimerin on tumor growth and tumor angiogenesis *in vivo*, we used a xenograft human breast cancer model and found that 3 mg/kg of pristimerin applied every other day significantly reduced both tumor volume (Figure 2A) and tumor weight (Figure 2B). As shown in Figure 2A, the tumors in the control group increased in size from 114.95 ± 27.50 to 501.06 ± 135.10 mm^3^, whereas the tumors in the pristimerin-treated group shrank from 115.76 ± 29.80 to 109.32 ± 54.40 mm^3^. Additionally, the average final tumor weight in the pristimerin-treated group was dramatically reduced compaired with that in the control group (Figure 2B), suggesting that pristimerin strongly inhibited tumor growth in this xenograft mouse breast tumor model. 

To investigate further whether pristimerin inhibited tumor angiogenesis in the xenograft mouse model, we stained solid tumor sections with a blood vessel staining kit. The mean number of blood vessels in the pristimerin-treated group was 37.06 ± 19.09/HPF compared with 100 ± 24.87/HPF in the control group (Figure 2D), indicating that pristimerin significantly inhibited tumor angiogenesis.

### 2.2. Pristimerin Inhibits Angiogenesis in Vivo and VEGF-induced Vessel Sprouting *ex Vivo*

**Figure 3:**
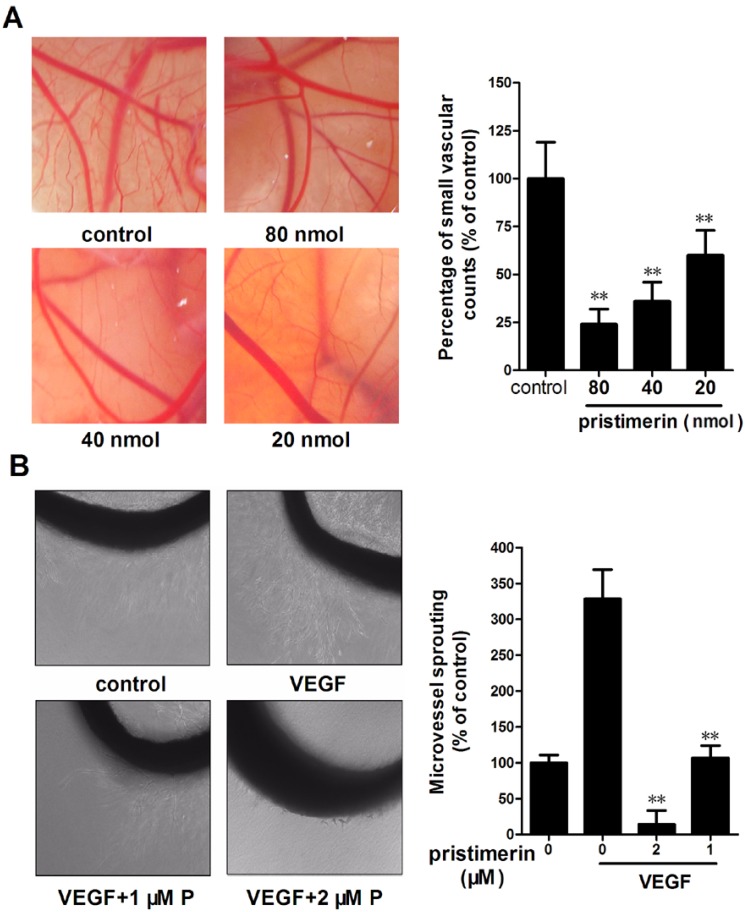
Pristimerin inhibits angiogenesis *in vivo* and VEGF-induced vessel sprouting *ex vivo.* (**A**) Effect of pristimerin on new blood vessel formation in CAMs. CAM were treated with pristimerin for 48 h, and then harvested and photographed. Quantification of neovascularization of the CAMs. Columns, mean; bars, SD. * *p* < 0.05 or ** *p* < 0.01 *versus* control; (**B**) Effect of pristimerin on microvessel sprouting in mouse aortic ring assay. Aortic segments isolated from Sprague-Dawley rats treated with VEGF (20 ng/mL) in the presence or absence of pristimerin for 7 d (magnification, ×100). P, pristimerin; Columns, mean; bars, SD. ** *p* < 0.01 *versus* VEGF alone.

We performed a chick embryo chorioallantoic membrane (CAM) assay to determine the antiangiogenic effects of pristimerin *in vivo*. As illustrated in Figure 3A, new blood vessels formed well on CAMs in the control group after a 2-day incubation, whereas incubation with pristimerin at 40 nmol/egg resulted in a notable inhibition, and pristimerin at 80 nmol/egg drastically impaired CAM neovascularization accompanied by a lack of prominent vessel networks. These results demonstrate that pristimerin suppresses angiogenesis in a CAM model.

The aortic ring assay mimics several stages in angiogenesis, including endothelial cell proliferation, migration, and tube formation and is widely used to evaluate the antiangiogenic effects of putative therapeutic compounds [14]. To further investigate whether pristimerin inhibited VEGF-induced angiogenesis *ex vivo*, we examined the sprouting of vessels from aortic rings in the absence or presence of pristimerin. VEGF (20 ng/mL) significantly stimulated microvessel sprouting around the aortic rings (Figure 3B). Treatment with pristimerin antagonized the VEGF-induced sprouting in a dose-dependent manner. Treatment with pristimerin at 1 μM yielded a notable suppression of microvessel formation *versus* the control (Figure 3A), and 2 μM of pristimerin completely blocked the VEGF-induced microvessel sprouting of rat aortic rings (Figure 3B). The presence of pristimerin resulted in decreasing capillary sprouting from the rat aorta rings.

### 2.3. Pristimerin Inhibits the VEGF-Induced Chemotactic Motility, Capillary Structure Formation and Proliferation of HUVECs *in Vitro*

**Figure 4:**
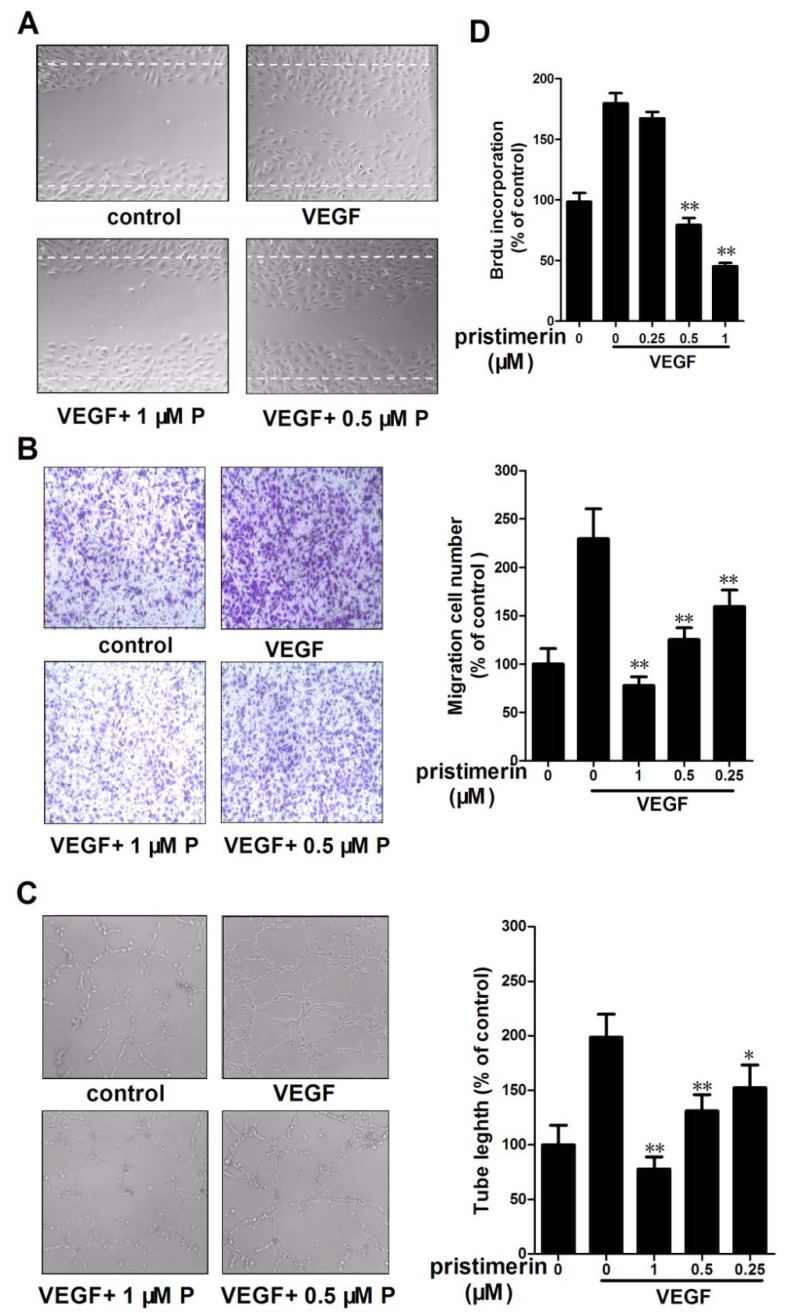
Pristimerin inhibits VEGF-induced chemotactic motility, capillary-structure formation and proliferation of endothelial cells. (**A**) pristimerin inhibited HUVEC migration. HUVECs were scratched by pipette and treated with or without 10 ng/mL VEGF and pristimerin(magnification, ×100). P, pristimerin; (**B**) Effect of pristimerin on VEGF-stimulated HUVECs migration in transwell assay. HUVECs were seeded in the upper chamber of a Transwell and treated with different concentrations of pristimerin. The bottom chamber was filled with ECGM supplemented with VEGF. After 8 h, the migrated cells were quantified by manual counting. (magnification, ×100). P, pristimerin; (**C**) pristimerin inhibits VEGF-induced capillary-structure formation of endothelial cells. HUVECs were placed in 96-well plates coated with Matrigel (2.0 × 10^4^ cells/well). After 6 h, tubular structures were photographed (magnification, ×100). P, pristimerin; (**D**) pristimerin inhibited the VEGF-induced cell proliferation of HUVECs. Columns, mean; bars, SD. ** *p* < 0.01 *versus* VEGF alone.

The migration of endothelial cells is essential for blood vessel formation in angiogenesis and thus for tumor growth. The effects of pristimerin on the chemotactic motility of HUVECs were assessed in a wound-healing migration assay and in a Transwell cell migration assay. As shown in Figure 4A, pristimerin significantly inhibited HUVEC migration in the wound-healing migration assay and dramatically reduced the VEGF-induced migration at 1 μM in the Transwell assay (Figure 4B). Pristimerin inhibits VEGF-induced HUVEC chemotactic motility in a concentration-dependent manner.

Tube formation by endothelial cells is one of the key steps of angiogenesis. We next evaluated the effects of pristimerin on tube formation by HUVECs in Matrigel assays. In this assay, HUVECs become elongated and form capillary-like structures on Matrigel [15]. Stimulation with 10 ng/mL VEGF promoted the differentiation of HUVECs to form robust tubular-like structures (Figure 4C). Pristimerin significantly inhibited the VEGF-induced tube formation by HUVECs on Matrigel at 0.5 μM, suggesting the potential effect of pristimerin on angiogenesis

Angiogenesis requires the proliferation of endothelial cells. We next examined the inhibitory effects of pristimerin on VEGF-induced HUVEC proliferation using a cell proliferation assay involving bromodeoxyuridine (BrdU) incorporation. As shown in Figure 3C, 0.5 μM pristimerin significantly decreased the VEGF-induced proliferation of HUVECs (Figure 4D). To determine if the effect of pristimerin on the tumor vasculature, we evaluated the MDA-MB-231 cell viability after 24 h of pristimerin treatment. Pristimerin at the same dose had less inhibitory effect on MDA-MB-231 cell viability (<5%). Pristimerin had higher inhibitory effect on endothelial cells than that on cancer cells. These results demonstrate that pristimerin blocks VEGF-induced *in vitro* angiogenesis by inhibiting cell proliferation, chemotactic motility and tube formation.

### 2.4. Influence of Pristimerin on VEGFR2 and Related Signaling Pathways

**Figure 5:**
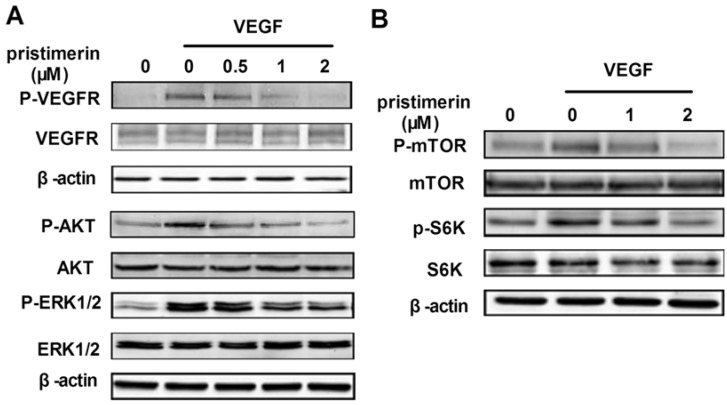
Pristimerin inhibits VEGFR2 kinase activity and its downstream signaling molecules. (**A**) pristimerin suppressed the activation of VEGFR2 and its downstream cascade. The activation of VEGFR2 and ERK/AKT from different treatments was tested by Western blotting and probed specific antibodies; (**B**) The mTOR signaling pathway were suppressed by pristimerin. Proteins from different treatments were analyzed by Western blotting and probed with specific antibodies. Three independent experiments were performed.

To determine the molecular basis of pristimerin-mediated antiangiogenesis, we examined the signaling molecules and pathways activated by the interaction of VEGFR2 with VEGF using Western blotting assays. Figure 5A illustrates how pristimerin inhibited VEGF-activated VEGFR2 phosphorylation in a dose-dependent manner. VEGFR2 activation leads to the activation of diverse intracellular signaling molecules that are responsible for endothelial cell migration, proliferation, and survival. To further investigate the intracellular signaling pathway affected by pristimerin, we screened some key kinases involved in angiogenesis signaling. We found that 0.5 and 1 μM of pristimerin significantly suppressed the activation of AKT and ERK1/2 (Figure 5A), respectively, and that 2 μM of pristimerin significantly inhibited the activation of mTOR and p70S6K (Figure 5B), which suggested that pristimerin exerted its antiangiogenic effects through the inhibition of VEGFR2 activation on the surfaces of endothelial cells and suppression of the AKT/mTOR/S6K kinase signaling pathway.

### 2.5. Discussion

In recent years, increasing effort has been focused on the identification of new anti-cancer compounds. Phytochemicals play an essential role in the search for new anticancer molecules; triterpenoids, possessing a wide range of unique biological activities, have received the most attention in this area [16,17,18]. Pristimerin is a triterpenoid isolated from Celastrus and Maytenus spp., and antitumor properties have also been reported for pristimerin [9,10,11,19]. In this study, we found that pristimerin displays potent antiangiogenic activities both *in vitro* and *in vivo*. Pristimerin inhibits tumor angiogenesis in a xenograft mouse breast tumor model, reducing the neovascularization of chicken chorioallantoic membrane *in vivo* and abrogating VEGF-induced microvessel sprouting in an *ex vivo* rat aortic ring assay. Pristimerin blocks VEGF-induced *in vitro* angiogenesis by inhibiting cell proliferation, chemotactic motility and tube formation. It is noteworthy that pristimerin at the same concentration exhibits a higher potency to suppress proliferation of activated endothelial cells (at angiogenic state) compared with that of cancer cells, suggesting pristimerin is a relatively affordable drug that specifically targets activated endothelial cells. Pristimerin exhibits a potent inhibition of angiogenesis. When angiogenesis was suppressed by pristimerin, tumor growth was substantially inhibited.

VEGF is the primary and the most potent inducer of angiogenesis. VEGF signaling events related to tumor growth and angiogenesis are mainly mediated by VEGFR2 [4]. VEGF-A and VEGFR2 play crucial roles in vessel sprouting and new vessel initiation through the induction of the proliferation, migration, and survival of endothelial cells [20]. We found that pristimerin significantly downregulated the phosphorylation of VEGFR2 induced by VEGF in a dose-dependent manner. VEGFR2 receptor tyrosine phosphorylation induced by binding with VEGF can stimulate intracellular signaling pathways, such as the MAPK/ERK pathway and the phosphatidylinositol 3-kinase/AKT pathway [21]. We found that pristimerin caused a downregulation of the VEGF-induced phosphorylation of both ERK1/2 and Akt, which are the key signaling molecules of these signaling pathways. ERK1/2 activation exerts its regulatory effects on the proliferation, differentiation and survival of endothelial cells [22], and the protein AKT is importantly involved in cell proliferation, survival and migration in endothelial cells [23]. Our results in the VEGF stimulation models *in vitro* showed that pristimerin inhibits multiple steps of angiogenesis including VEGF-induced cell proliferation, motility, and tube formation. The inhibitory effect of pristimerin on the cell proliferation, motility, and tube formation of HUVECs appeared to be associated with its ability to suppress the phosphorylation of VEGFR2 and ERK1/2 and Akt. Our results demonstrate that pristimerin potently inhibits angiogenesis by significantly inhibiting VEGFR2 activation.

Notably, pristimerin has a more potent inhibitory effect on phosphorylation of Akt than on the phosphorylation of VEGFR2. This highly inhibitory effect suggests that pristimerin may also affect other pathway involved in regulating angiogenesis. Recently, the Akt/mTOR/P70S6K signaling pathway has been identified as a novel functional target in angiogenesis [6,18,24]. mTOR is critical for cellular proliferation and growth in endothelial cells [25]. The S6K1 (ribosomal p70S6 kinase) protein is mainly a downstream effector of mTORC1, leading to the initiation of translation and thereby increasing protein synthesis [26], additionally regulating cell migration by inducing actin filament remodeling to form filopodia and lamellipodia structures [27]. Our present data demonstrate that pristimerin significantly inhibits the activation of AKT/mTOR pathway in endothelial cells, including AKT, mTOR kinase and its downstream ribosomal S6 kinase, in a concentration-dependent manner. This inhibitory effect of pristimerin on the AKT/mTOR/P70S6K signaling pathway may, at least in part, contribute to the inhibition of endothelial cell proliferation, migration, and capillary structure formation. Thus, the antiangiogenic properties of pristimerin may be partially due to its inhibition of the AKT/mTOR/P70S6K signaling pathway.

Importantly, our study revealed that pristimerin decreased the VEGF secretion of MDA-MB-231 cells and had no effect on MDA-MB-231 cell viability at the same concentration (data not shown). Pristimerin exerts inhibitory effects on NFκB activation [9,28], which is known to regulate the expression of angiogenic gene products; Additionally, the mTOR/p70S6K pathway has been found to regulate the expression of hypoxia-inducible factor-1 α to influence the secretion of proangiogenic factors in various human carcinomas [29,30,31]. Previous studies have examined the effects of mTORC1/mTORC2 inhibition on VEGF secretion *in vitro* and *in vivo* [6,31]. All the above mechanisms might contribute to this compound’s antiangiogenic properties in the xenograft mouse tumor model. We are interested in investigating the mechanism of the decreased secretion of proangiogenic molecules (VEGF) by pristimerin. 

## 3. Experimental

### 3.1. Cell lines and Cell Culture

Primary human umbilical vascular endothelial cells (HUVECs) were isolated from human umbilical cord veins by collagenase treatment as described previously [14]. The HUVECs were cultured in endothelial cell growth medium (ECGM): M199 medium (Gibco-BRL, Grand Island, NY, USA) supplemented with 20 μg/mL bovine endothelial cell growth factor (Roche Molecular Biochemicals, Laval, Quebec, Canada), penicillin (50 IU/L), streptomycin (50 mg/L), 50 μg/mL amphotericin B, 0.1 mg/mL heparin (Sigma, St. Louis, MO, USA), 15 mmol/L HEPES buffer, NaHCO_3_ (44 mmol/L) and 20% fetal bovine serum (FBS, Gibco-BRL) at 37 °C under a humidified 95%:5% (v/v) mixture of air and CO_2_, as described previously [32]. Human breast cancer (MDA-MB-231) cells were purchased from the American Type Culture Collection and cultured in Leibovitz's L-15 medium (Gibco-BRL). supplemented with 10% FBS (Gibco-BRL) at 37 °C under a humidified 100% air atmosphere.

### 3.2. Reagents and Antibodies

A 10 mM stock solution of pristimerin (purchased from Enzo Life Sciences, Lausen, Switzerland, purity ≥98%) was stored at −20 °C in small aliquots protected from light and was diluted to the required concentrations in cell culture medium. Recombinant human VEGF 165 was purchased from R&D Systems (Minneapolis, MN, USA), and growth factor-reduced Matrigel was purchased from BD Biosciences (Bedford, MA, USA). The antibodies against β-actin were obtained from Santa Cruz Biotechnology (Santa Cruz, CA, USA). The anti-AKT, anti-ERK1/2, anti-VEGFR-2, anti-mTOR, anti-p70S6K1, and phosphorylated-specific anti-AKT (Ser473), anti-ERK1/2 (Thr202/Tyr204), anti-VEGFR2 (Tyr1175), anti-mTOR (Ser2448), and anti-p70S6K1 (Thr389) antibodies were purchased from Cell Signaling Technology (Danvers, MA, USA).

### 3.3. Human Breast Tumor Xenograft Mouse Model

The experimental protocol was established according to the NIH guidelines for Animal Research and Care and was approved by the Institutional Animal Care and Use Committee. A xenograft human breast cancer mouse model assay was performed as previously described [33]. Six-week-old female BALB/c nude mice (National Rodent Laboratory Animal Resources, Shanghai, China) weighing approximately 22 g were randomly divided into two groups of six each. MDA-MB-231 cells were s.c. injected into the mice (8 × 10^6^ per mouse). After the tumors grew to approximately 100 mm^3^, the mice were injected subcutaneously with either vehicle or pristimerin (3 mg/kg) every other day for 16 days. The tumor size was measured with a caliper (calculated volume = shortest diameter^2^ × longest diameter/2) at two day intervals.

### 3.4. Histology and Immunohistochemistry

Solid tumors were removed and fixed with 10% formaldehyde and embedded in paraffin. Specific blood vessel staining was performed on 5-μm deparaffinized sections with a specific blood vessel staining kit (von Willebrand Factor; Millipore, Billerica, MA, USA). Section images were obtained with using a Leica DM 4000B photo microscope (Solms, Germany) at a magnification of 200×. Blood vessels were counted (n = 5).

### 3.5. Chicken Chorioallantoic Membrane (CAM) Assay

The antiangiogenic activity of pristimerin on CAM was assayed as described previously [17]. Briefly, fertilized chicken eggs were incubated at 37 °C and 60%–70% relative humidity for 6 days. After this incubation, a small hole was punched into the broad side of the egg, and a window was carefully created through the egg shell. Sterilized filter paper disks (5 × 5 mm) saturated with either vehicle or pristimerin (20, 40, or 80 nmol/egg) were placed on the CAMs. The window was sealed with cellophane tape, and the eggs were replaced in the incubator and maintained at 37 °C for another 2 days. Finally, the sterilized filter paper disks were removed, after which the CAMs were photographed. Ten eggs were used per group, and the number of newly formed vessels was counted. The angiogenic index was defined as the mean number of visible blood vessel branches, the percentage of inhibition was expressed relative to untreated wells at 100%. Three independent experiments were performed in each trial.

### 3.5. Rat Aortic Ring Assay

The rat aortic ring assay was performed as described previously [14]. Thoracic aortas isolated from 6-week-old Sprague-Dawley rats were cleaned of periadventitial fat and connective tissues and cut into 1- to 1.5-mm-thick rings. After being rinsed five times with M199, these rings were placed in 24-well plates. The clotting medium contained M199+ (M199 with 100 U/mL penicillin and 100 μg/mL streptomycin) plus 0.3% fibrinogen and 0.5% ε-amino-*n*-caproic acid (ACA, Sigma). The growth medium consisted of M199+ with 0.5% FBS and 0.5% ACA. Next, 20 ng/mL VEGF in 1 mL of M199+ with or without various concentrations of pristimerin were added to the wells. As a control, medium alone was assayed. After 7 days, microvessel growth was quantified by manually counting the number of microvessels sprouting from the rat aortic rings, with six rings being used as a group. The percentage of inhibition was expressed relative to untreated wells at 100%. The experimental protocol was established according to the NIH guidelines for Animal Research and Care and was approved by the Institutional Animal Care and Use Committee. Three independent experiments were performed in each trial.

### 3.7. Cell Proliferation Assay by Bromodeoxyuridine (BrdU) Incorporation

Cell proliferation was measured using a BrdU ELISA kit (Roche Applied Science, Mannheim, Germany) according to the manufacturer’s instructions [34]. HUVECs (5 × 10^3^ cells/well) were plated in 96-well plates. After 24 h, the cells were first synchronized in serum-free ECGM containing 0.5% FBS for 6 h and cultured in 100 μL of serum-free ECGM containing various concentrations of pristimerin with or without VEGF for 24 h at 37 °C. The cells were incubated with 20 μL of a BrdU-labeling solution per well for 4 h and were dried, fixed, and detected using anti-BrdU mAb. Finally, the BrdU incorporation was determined by measuring the optical density at a wavelength of 450 nm using a reference wavelength of 690 nm. Triplicate wells were analyzed for each concentration, and the BrdU assays were repeated in triplicate.

### 3.8. Wound-Healing Migration Assay

HUVECs were allowed to grow to full confluence in 6-well plates precoated with 0.1% gelatin (Sigma) and were subsequently starved with serum-free ECGM containing 0.5% FBS for 6 h to inactivate cell proliferation. The cells were wounded with pipette tips and washed with phosphate-buffered saline. Serum-free ECGM containing 0.5% FBS was added to the wells with or without 10 ng/mL VEGF and various concentrations of pristimerin. Images of the cells were recorded after 8 h of incubation at 37 °C in a 95%:5% (v/v) mixture of air and CO2. Three independent experiments were performed. 

### 3.9. Transwell Migration Assay

The chemotactic motility of the HUVECs was determined using a Transwell migration assay (BD Biosciences) as described previously [18]. Briefly, the bottom chambers were filled with 600 μL of serum-free ECGM containing 0.5% FBS supplemented with 10 ng/mL VEGF. The top chambers were seeded with inactivated HUVECs (1.8 × 10^4^ cells) suspended in 100 μL of serum-free ECGM containing 0.5% FBS plus various concentrations of pristimerin. After 8 h of migration, the non-migrated cells were removed with cotton swabs, and the migrated cells were fixed with cold 4% paraformaldehyde and stained with 1% crystal violet. Images were recorded (Olympus; magnification, ×100). The migrated cells were counted manually, and the percentage of inhibition was expressed in comparison with the untreated control wells at 100%. Three independent experiments were performed.

### 3.10. Capillary-Like Tube Formation Assay

Tube formation was assessed as previously described [35]. Growth factor–reduced Matrigel was pipetted into pre-chilled 96-well plates (60 μL Matrigel/well) and polymerized for 60 min at 37 °C. HUVECs were first incubated in serum-free ECGM containing 0.5% FBS for 6 h, subsequently pretreated with various dilutions of pristimerin for 30 min, and finally seeded onto the Matrigel layer in 96-well plates at a density of 2.0 × 10^4^ cells/well. Serum-free ECGM containing 0.5% FBS was added with or without 10 ng/mL VEGF. After 6 h of incubation at 37 °C in a 95%:5% (v/v) mixture of air and CO2, the tubular structures of the endothelial cells were photographed using an inverted microscope (Olympus; magnification, ×100). The tube length of the capillary-like structure was calculated randomly from five fields. Inhibition percentage was expressed using untreated wells as 100%. Three independent experiments were performed.

### 3.11. Western Immunoblot Analysis

To determine the signaling mechanism of pristimerin involved in angiogenesis, HUVECs were first starved in serum-free ECGM for 6 h and were pretreated with or without pristimerin for 60 min followed by stimulation with 50 ng/mL of VEGF for 2 min (for VEGFR2 activation) or 20 min (to activate signaling downstream of VEGFR2). After stimulation, the cells were harvested and lysed followed by centrifugation. The protein concentrations of the supernatants were measured with the BCA (bicinchoninic acid) assay using a Varioskan multimode microplate spectrophotometer (Thermo, Waltham, MA, USA). About 30–40 μg of cellular protein from each sample was applied to 8–12% SDS-polyacrylamide gels and probed with specific antibodies followed by exposure to horseradish peroxidase-conjugated goat anti-mouse or goat anti-rabbit antibodies. 

### 3.12. Statistical Analysis

All of the data in the different experimental groups were expressed as means ± SD. The data reported herein were obtained in at least three independent experiments. Statistical comparisons between the treated groups and the untreated group were performed by one-way analysis of variance (ANOVA) followed by Dunnet’s test, the difference between two groups was analyzed by Student’s *t*-test. A P value of <0.05 was considered statistically significant. 

## 4. Conclusions

In conclusion, our results demonstrate that pristimerin potently suppresses angiogenesis *in vitro* and *in vivo* by targeting VEGFR2 activation, leading to the suppression of tumor growth. Our discovery suggests that pristimerin has great potential as an anti-cancer therapeutic agent.

## References

[1] Weis S.M., Cheresh D.A. (2011). Tumor angiogenesis: Molecular pathways and therapeutic targets. Nat. Med..

[2] Carmeliet P., Jain R.K. (2000). Angiogenesis in cancer and other diseases. Nature.

[3] Kerbel R.S. (2008). Tumor angiogenesis. N. Engl. J. Med..

[4] Kowanetz M., Ferrara N. (2006). Vascular endothelial growth factor signaling pathways: Therapeutic perspective. Clin. Cancer Res..

[5] Pyun B.J., Choi S., Lee Y., Kim T.W., Min J.K., Kim Y., Kim B.D., Kim J.H., Kim T.Y., Kim Y.M. (2008). Capsiate, a nonpungent capsaicin-like compound, inhibits angiogenesis and vascular permeability via a direct inhibition of Src kinase activity. Cancer Res..

[6] Falcon B.L., Barr S., Gokhale P.C., Chou J., Fogarty J., Depeille P., Miglarese M., Epstein D.M., McDonald D.M. (2011). Reduced VEGF production, angiogenesis, and vascular regrowth contribute to the antitumor properties of dual mTORC1/mTORC2 inhibitors. Cancer Res..

[7] Burstein H.J., Schwartz R.S. (2008). Molecular origins of cancer. N. Engl. J. Med..

[8] Costa P.M., Ferreira P.M., Bolzani Vda S., Furlan M., de Freitas Formenton Macedo Dos Santos V.A., Corsino J., de Moraes M.O., Costa-Lotufo L.V., Montenegro R.C., Pessoa C. (2008). Antiproliferative activity of pristimerin isolated from Maytenus ilicifolia (Celastraceae) in human HL-60 cells. Toxicol. In Vitro.

[9] Tiedemann R.E., Schmidt J., Keats J.J., Shi C.X., Zhu Y.X., Palmer S.E., Mao X., Schimmer A.D., Stewart A.K. (2009). Blood.

[10] Byun J.Y., Kim M.J., Eum D.Y., Yoon C.H., Seo W.D., Park K.H., Hyun J.W., Lee Y.S., Lee J.S., Yoon M.Y. (2009). Reactive oxygen species-dependent activation of Bax and poly(ADP-ribose) polymerase-1 is required for mitochondrial cell death induced by triterpenoid pristimerin in human cervical cancer cells. Mol. Pharmacol..

[11] Yang H., Landis-Piwowar K.R., Lu D., Yuan P., Li L., Reddy G.P., Yuan X., Dou Q.P. (2008). Pristimerin induces apoptosis by targeting the proteasome in prostate cancer cells. J. Cell Biochem..

[12] Boire A., Covic L., Agarwal A., Jacques S., Sherifi S., Kuliopulos A. (2005). PAR1 is a matrix metalloprotease-1 receptor that promotes invasion and tumorigenesis of breast cancer cells. Cell.

[13] Wu C.C., Chan M.L., Chen W.Y., Tsai C.Y., Chang F.R., Wu Y.C. (2005). Pristimerin induces caspase-dependent apoptosis in MDA-MB-231 cells via direct effects on mitochondria. Mol. Cancer Ther..

[14] Chen Y., Lu N., Ling Y., Wang L., You Q., Li Z., Guo Q. (2010). J. Pharmacol. Sci..

[15] Taraboletti G., Giavazzi R. (2004). Modelling approaches for angiogenesis. Eur. J. Cancer.

[16] Sheng H., Sun H. (2011). Synthesis, biology and clinical significance of pentacyclic triterpenes: A multi-target approach to prevention and treatment of metabolic and vascular diseases. Nat. Prod. Rep..

[17] Salminen A., Lehtonen M., Suuronen T., Kaarniranta K., Huuskonen J. (2008). Terpenoids: Natural inhibitors of NF-kappaB signaling with anti-inflammatory and anticancer potential. Cell Mol. Life Sci..

[18] Pang X., Yi Z., Zhang J., Lu B., Sung B., Qu W., Aggarwal B.B., Liu M. (2010). Celastrol suppresses angiogenesis-mediated tumor growth through inhibition of AKT/mammalian target of rapamycin pathway. Cancer Res..

[19] Yadav V.R., Prasad S., Sung B., Kannappan R., Aggarwal B.B.  (2010). Targeting inflammatory pathways by triterpenoids for prevention and treatment of cancer. Toxins (Basel).

[20] Napoleone Ferrara R.S.K. (2005). Angiogenesis as a therapeutic target. Nature.

[21] Holmes K., Roberts O.L., Thomas A.M., Cross M.J. (2007). Vascular endothelial growth factor receptor-2: Structure, function, intracellular signalling and therapeutic inhibition. Cell. Signal..

[22] Huang D., Ding Y., Luo W.M., Bender S., Qian C.N., Kort E., Zhang Z.F., VandenBeldt K., Duesbery N.S., Resau J.H., Teh B.T. (2008). Inhibition of MAPK kinase signaling pathways suppressed renal cell carcinoma growth and angiogenesis *in vivo*. Cancer Res..

[23] Hers I., Vincent E.E., Tavare J.M. (2011). Akt signalling in health and disease. Cell Signal..

[24] Pang X., Zhang L., Wu Y., Lin L., Li J., Qu W., Safe S., Liu M. (2010). Methyl 2-cyano-3,11-dioxo-18-olean-1,12-dien-30-oate (CDODA-Me), a derivative of glycyrrhetinic acid, functions as a potent angiogenesis inhibitor. J. Pharmacol. Exp. Ther..

[25] Lee D.F., Kuo H.P., Chen C.T., Hsu J.M., Chou C.K., Wei Y., Sun H.L., Li L.Y., Ping B., Huang W.C. (2007). IKK beta suppression of TSC1 links inflammation and tumor angiogenesis via the mTOR pathway. Cell.

[26] LoPiccolo J., Blumenthal G.M., Bernstein W.B., Dennis P.A. (2008). Targeting the PI3K/Akt/mTOR pathway: Effective combinations and clinical considerations. Drug Resist. Updat..

[27] Berven L.A., Willard F.S., Crouch M.F. (2004). Role of the p70(S6K) pathway in regulating the actin cytoskeleton and cell migration. Exp. Cell Res..

[28] Lu Z., Jin Y., Chen C., Li J., Cao Q., Pan J. (2010). Pristimerin induces apoptosis in imatinib-resistant chronic myelogenous leukemia cells harboring T315I mutation by blocking NF-kappaB signaling and depleting Bcr-Abl. Mol. Cancer.

[29] Garcia-Maceira P., Mateo J. (2009). Silibinin inhibits hypoxia-inducible factor-1alpha and mTOR/p70S6K/4E-BP1 signalling pathway in human cervical and hepatoma cancer cells: implications for anticancer therapy. Oncogene.

[30] Weng Q., Zhang J., Cao J., Xia Q., Wang D., Hu Y., Sheng R., Wu H., Zhu D., Zhu H., He Q., Yang B. (2011). Q39, a quinoxaline 1,4-Di-N-oxide derivative, inhibits hypoxia-inducible factor-1alpha expression and the Akt/mTOR/4E-BP1 signaling pathway in human hepatoma cells. Invest. New Drugs.

[31] Yu K., Toral-Barza L., Shi C., Zhang W.G., Lucas J., Shor B., Kim J., Verheijen J., Curran K., Malwitz D.J. (2009). Cancer Res..

[32] Pang X., Yi T., Yi Z., Cho S.G., Qu W., Pinkaew D., Fujise K., Liu M. (2009). Morelloflavone, a biflavonoid, inhibits tumor angiogenesis by targeting rho GTPases and extracellular signal-regulated kinase signaling pathway. Cancer Res..

[33] Inao T., Harashima N., Monma H., Okano S., Itakura M., Tanaka T., Tajima Y., Harada M.  (2011). Antitumor effects of cytoplasmic delivery of an innate adjuvant receptor ligand, poly(I:C), on human breast cancer. Breast Cancer Res. Treat..

[34] Abdel-Malak N.A., Mofarrahi M., Mayaki D., Khachigian L.M., Hussain S.N. (2009). Early growth response-1 regulates angiopoietin-1-induced endothelial cell proliferation, migration, and differentiation. Arterioscler. Thromb. Vasc. Biol..

[35] Pisanti S., Picardi P., Prota L., Proto M.C., Laezza C., McGuire P.G., Morbidelli L., Gazzerro P., Ziche M., Das A., Bifulco M. (2011). Genetic and pharmacologic inactivation of cannabinoid CB1 receptor inhibits angiogenesis. Blood.

